# Enhanced store-operated Ca^2+^ influx and ORAI1 expression in ventricular fibroblasts from human failing heart

**DOI:** 10.1242/bio.022632

**Published:** 2017-01-26

**Authors:** Gracious R. Ross, Tanvir Bajwa, Stacie Edwards, Larisa Emelyanova, Farhan Rizvi, Ekhson L. Holmuhamedov, Paul Werner, Francis X. Downey, A. Jamil Tajik, Arshad Jahangir

**Affiliations:** 1 Center for Integrative Research on Cardiovascular Aging, Aurora Research Institute, Aurora Health Care, Milwaukee, WI 53215, USA; 2 Aurora Cardiovascular Services, Aurora Sinai/Aurora St. Luke's Medical Centers, Milwaukee, WI 53215, USA

**Keywords:** Calcium, Cardiac fibrosis, Store-operated Ca^2+^ entry, Heart failure

## Abstract

Excessive cardiac fibrosis, characterized by increased collagen-rich extracellular matrix (ECM) deposition, is a major predisposing factor for mechanical and electrical dysfunction in heart failure (HF). The human ventricular fibroblast (hVF) remodeling mechanisms that cause excessive collagen deposition in HF are unclear, although reports suggest a role for intracellular free Ca^2+^ in fibrosis. Therefore, we determined the association of differences in cellular Ca^2+^ dynamics and collagen secretion/deposition between hVFs from failing and normal (control) hearts. Histology of left ventricle sections (Masson trichrome) confirmed excessive fibrosis in HF versus normal. *In vitro*, hVFs from HF showed increased secretion/deposition of soluble collagen in 48 h of culture compared with control [85.9±7.4 µg/10^6^ cells vs 58.5±8.8 µg/10^6^ cells, *P*<0.05; (Sircol™ assay)]. However, collagen gene expressions (*COL1A1* and *COL1A2*; RT-PCR) were not different. Ca^2+^ imaging (fluo-3) of isolated hVFs showed no difference in the thapsigargin-induced intracellular Ca^2+^ release capacity (control 16±1.4% vs HF 17±1.1%); however, Ca^2+^ influx via store-operated Ca^2+^ entry/Ca^2+^ release-activated channels (SOCE/CRAC) was significantly (*P*≤0.05) greater in HF-hVFs (47±3%) compared with non-failing (35±5%). Immunoblotting for *I*_CRAC_ channel components showed increased ORAI1 expression in HF-hVFs compared with normal without any difference in STIM1 expression. The Pearson's correlation coefficient for co-localization of STIM1/ORAI1 was significantly (*P*<0.01) greater in HF (0.5±0.01) than control (0.4±0.01) hVFs. The increase in collagen secretion of HF versus control hVFs was eliminated by incubation of hVFs with YM58483 (10 µM), a selective I_CRAC_ inhibitor, for 48 h (66.78±5.87 µg/10^6^ cells vs 55.81±7.09 µg/10^6^ cells, *P*=0.27). In conclusion, hVFs from HF have increased collagen secretion capacity versus non-failing hearts and this is related to increase in Ca^2+^ entry via SOCE and enhanced expression of ORAI, the pore-forming subunit. Therapeutic inhibition of SOCE may reduce the progression of cardiac fibrosis/HF.

## Abstract (precis)

**Summary:** The excessive collagen secretory phenotype found in failing human hearts is associated with ventricular fibroblast remodeling, caused by an elevated influx of intracellular calcium via SOC channels.

## INTRODUCTION

Excessive cardiac fibrosis is a major predisposing factor for mechanical and electrical dysfunction in heart failure (HF) (Ichiki et al., 2014). Fibrosis is characterized by the progressive replacement of normal parenchymal tissue with collagen-rich extracellular matrix (ECM) (Desmoulière et al., 2003; Liu et al., 2010). Despite a known association of fibrosis with progressive myocardial dysfunction, the mechanisms underlying enhanced ECM deposition are not fully understood and no effective therapy is available to limit progression of fibrosis in patients with HF (Ichiki et al., 2014). Fibroblasts are the most abundant cells in the heart and contribute to fibrosis (Hinz, 2015). The fibrogenic processes that are critical for the progression of fibrosis, including excessive fibroblast proliferation, activation, and secretion of extracellular matrix and cytokines, are dependent on intracellular Ca^2+^ (Clapham, 2007; Zhang et al., 2014). Hence, defining regulatory pathways that increase Ca^2+^ mobilization in fibroblasts from HF patients could identify novel targets that can limit fibrosis and HF progression.

Since intracellular Ca^2+^ release and store-operated Ca^2+^ entry (SOCE) are the major intracellular Ca^2+^ sources in nonexcitable cells, including fibroblasts, any possible upregulation of either source in the failing heart could contribute to excessive fibroblast proliferation, activation, and ECM deposition. Therefore, the objective of this study was to determine the differences in Ca^2+^ dynamics and collagen secretion/deposition between human ventricular fibroblasts (hVF) from failing and normal hearts. Specifically, we investigated the extent of intracellular Ca^2+^ release; store-operated channels (SOC) mediated Ca^2+^ influx, particularly the contribution of the I_CRAC_ channel to regulation of intracellular free Ca^2+^ in fibroblasts; the expression of the pore-forming ORAI1 and the store Ca^2+^ sensor, STIM1; magnitude of collagen secretion/deposition; and whether selective inhibition of the I_CRAC_ channel could mitigate the altered collagen secretion/deposition, if any, in ventricular fibroblasts from HF patients.

## RESULTS

### Histological evidence for increased ventricular fibrosis in the failing heart

**Fig. 1.**
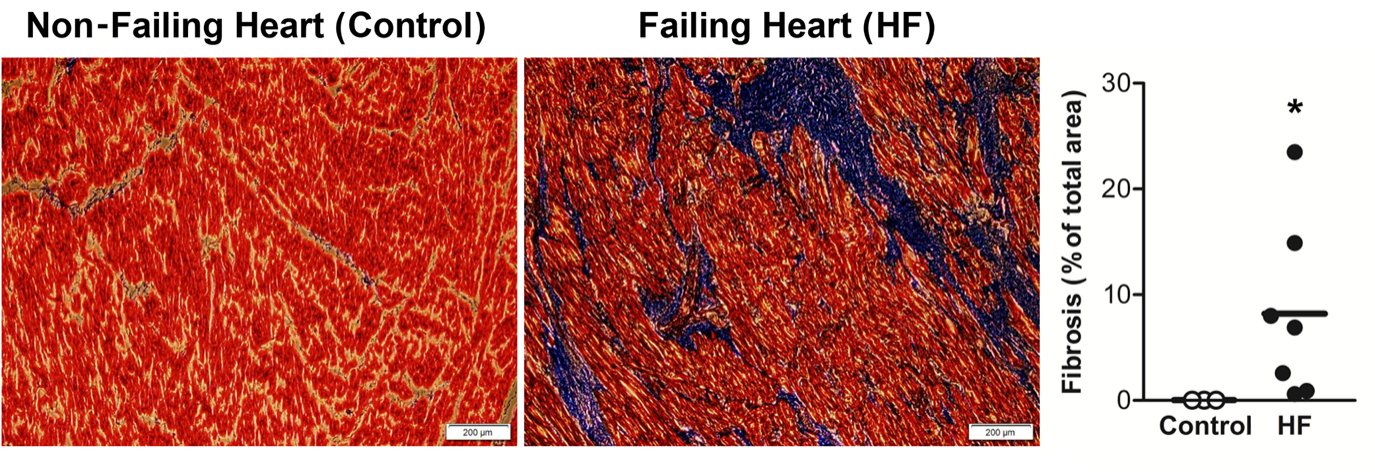
**Significant fibrosis in left ventricle of human failing heart.** Representative photographs of Masson's trichrome staining of left ventricle histology sections display severe fibrosis (blue) in the failing heart (right) compared with the normal heart (left). The individual data points column graph on the right displays fibrosis area as a percentage of total area (mean line and each sample value), depicting a significantly higher percentage of blue area (fibrosis) in the failing heart as quantified by an ImageJ macro. Data were analyzed by unpaired *t*-test (one-tailed); *P≤0.05; control *n*=3, HF *n*=7.

We confirmed that human HF is associated with increased ventricular fibrosis. Left ventricle (LV) sections from non-failing and failing hearts were stained with Masson's trichrome to determine the secreted fibrillar collagens as a marker of fibrosis (Cleutjens et al., 1995). As shown in Fig. 1, LV sections from failing hearts had significantly greater fibrosis (blue) than control sections. The percentage of blue area, measured using ImageJ (NIH) macro, was significantly higher in failing hearts than normal hearts (mean±s.e.m., 8.2±3% vs 0.1±0.01%, *P*<0.05; control *n*=3, HF *n*=7).

### Enhanced collagen secretion from failing heart ventricular fibroblasts

**Fig. 2.**
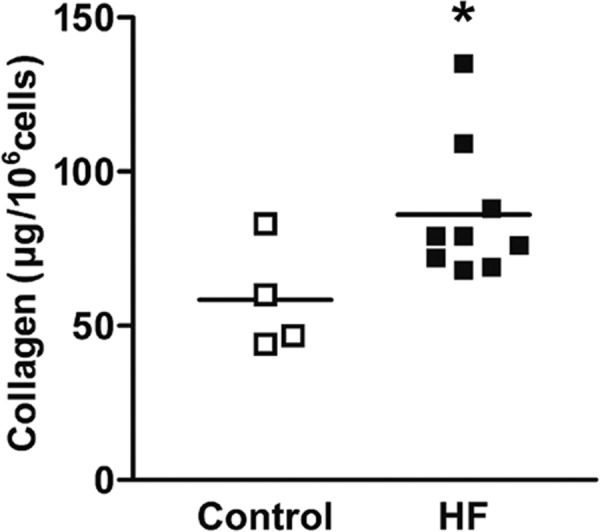
**Increased secretion/deposition of collagen by ventricular fibroblasts isolated from human failing hearts.** Collagen secretion/deposition was measured by Sircol soluble collagen assay in ventricular fibroblasts from normal and failing hearts after 48-h culture. The individual data points column graph displays each sample value of collagen secreted/deposited for each group with mean value line showing significantly greater collagen secretion in the failing heart group. Data were analyzed by unpaired *t*-test (two-tailed); *P≤0.05; control *n*=4, HF *n*=8.

The capacity of fibroblasts to synthesize and secrete precursors of fibrillar collagen, the major structural protein of the ECM, changes with cardiac remodeling (Lopez et al., 2010). Because excessive collagen secretion by fibroblasts can result in disproportionate fibrosis, we sought to determine whether there is any difference in collagen secretion capacity of ventricular fibroblasts between non-failing and failing human hearts. The amount of soluble collagen secreted and deposited during a 48-h period culture of both types of ventricular fibroblasts was assessed by Sircol™ soluble collagen assay kit (Biocolor Ltd., Carrickfergus, Northern Ireland, UK). The overall collagen secretion from human ventricular fibroblasts from failing hearts (hVF-HF) was 47% higher than non-failing hearts (control) (85.9±7.4 µg/10^6^ vs 58.5±8.8 µg/10^6^ cells, *P*<0.05; control *n*=4, HF *n*=9; Fig. 2).

### *COL1A* gene expression is not different between ventricular fibroblasts from normal and failing hearts

**Fig. 3.**
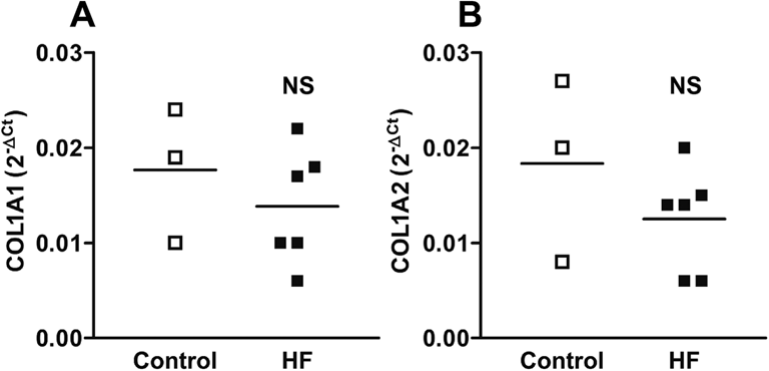
**Gene expression of collagen genes *COL1A1* and *COL1A2* in ventricular fibroblasts.** The individual data points column graphs display the mean (line) and each sample value of gene expression (2^−ΔCt^) of either *COL1A1* (A) or *COL1A2* (B) with no difference between the groups at mRNA levels. The real time-PCR was performed in total RNA isolated from hVFs after one day of culture. Horizontal bar represents mean value, analyzed by unpaired *t*-test (two-tailed); NS, not significant; *n*=3 per group.

The increased collagen secretion in hVF-HF could be the result of enhanced transcription of the collagen gene, translation to protein, or secretion of synthesized protein. To assess whether hVF-HFs have greater collagen secretion due to changes in transcription of collagen genes, real time-polymerase chain reaction (RT-PCR) for *COL1A1* and *COL1A2* gene expressions was performed and compared between the hVF-HF and control groups. As displayed in Fig. 3, there was no significant difference in expression of genes encoding *COL1A1* and *COL1A2* between human failing heart and non-failing heart ventricular fibroblasts.

### Enhanced store-operated Ca^2+^ influx in ventricular fibroblasts from failing hearts

**Fig. 4.**
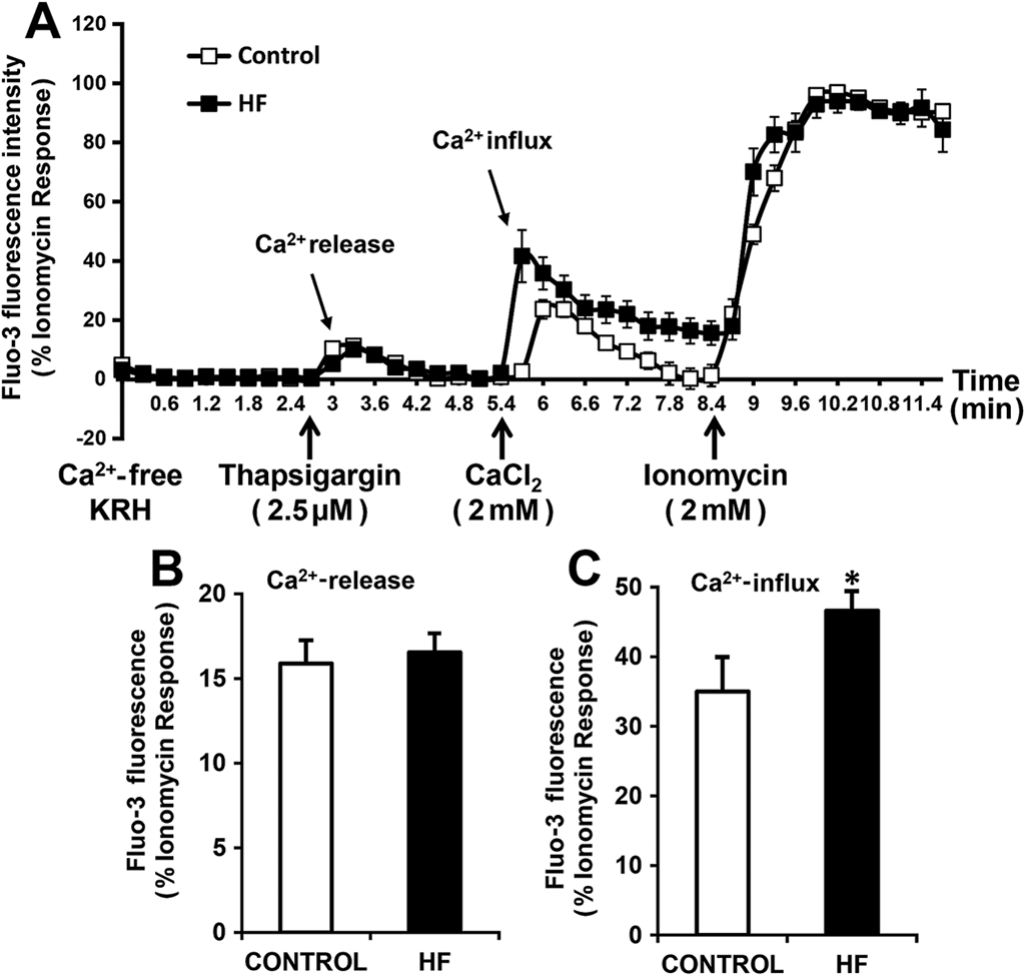
**Enhanced SOC Ca^2+^ entry in ventricular fibroblasts from failing hearts.** (A) A representative recording of intracellular free Ca^2+^ using confocal microscopy. Fibroblasts were loaded with fluo-3 dye and kept in Ca^2+^-free KRH solution. Thapsigargin (2.5 µM), a Ca^2+^-ATPase inhibitor, was used to deplete the store Ca^2+^ to open the plasma membrane store-operated channels (SOC), and the addition of external CaCl_2_ caused Ca^2+^ influx via SOC channels. (B,C) The bar graphs display the pooled average peak values for Ca^2+^ release from intracellular stores (B) and Ca^2+^ influx via SOCE (C). While there was no difference in the intracellular Ca^2+^ release (B) between the groups, the human ventricular fibroblasts from failing hearts showed significantly increased Ca^2+^ influx (C) compared with ventricular fibroblasts from normal hearts. Data are presented as mean±s.e.m, analyzed by unpaired *t*-test (two-tailed); *P≤0.05; control *n*=6, HF *n*=15.

Since the message at mRNA level for collagen synthesis was not increased in hVF-HF, the signaling mechanism that triggers increased secretion – specifically, the involvement of intracellular Ca^2+^ – was assessed, as Ca^2+^ is an important second messenger that is involved in many of the fibroblast functions, including secretion and other receptor-mediated signal transduction mechanisms (Clapham, 2007). In non-excitable cells like fibroblasts, the major sources of Ca^2+^ are the intracellular Ca^2+^ stores and the entry via SOC (Chen et al., 2010; Davis et al., 2012). Therefore, we determined the magnitude of Ca^2+^ release from intracellular stores and Ca^2+^ influx via SOC channels and compared between hVF-HF and control after normalizing to maximum Ca^2+^ influx (being considered as 100%) induced by the Ca^2+^ ionophore ionomycin (2 µM), as shown in Fig. 4A. There was no difference in the thapsigargin-induced intracellular Ca^2+^ release capacity between control and hVF-HF (16±1.4% vs 16.5±1.1% , *P*=0.7; control *n*=6, hVF-HF *n*=15; Fig. 4B), but Ca^2+^ influx via the SOC channels was significantly (*P*≤0.05) greater in the hVF-HF group (47±3%; *n*=15) than the control group (35±5%; *n*=6; Fig. 4C).

### ORAI1, but not STIM1, expression is elevated in ventricular fibroblasts from failing hearts

**Fig. 5.**
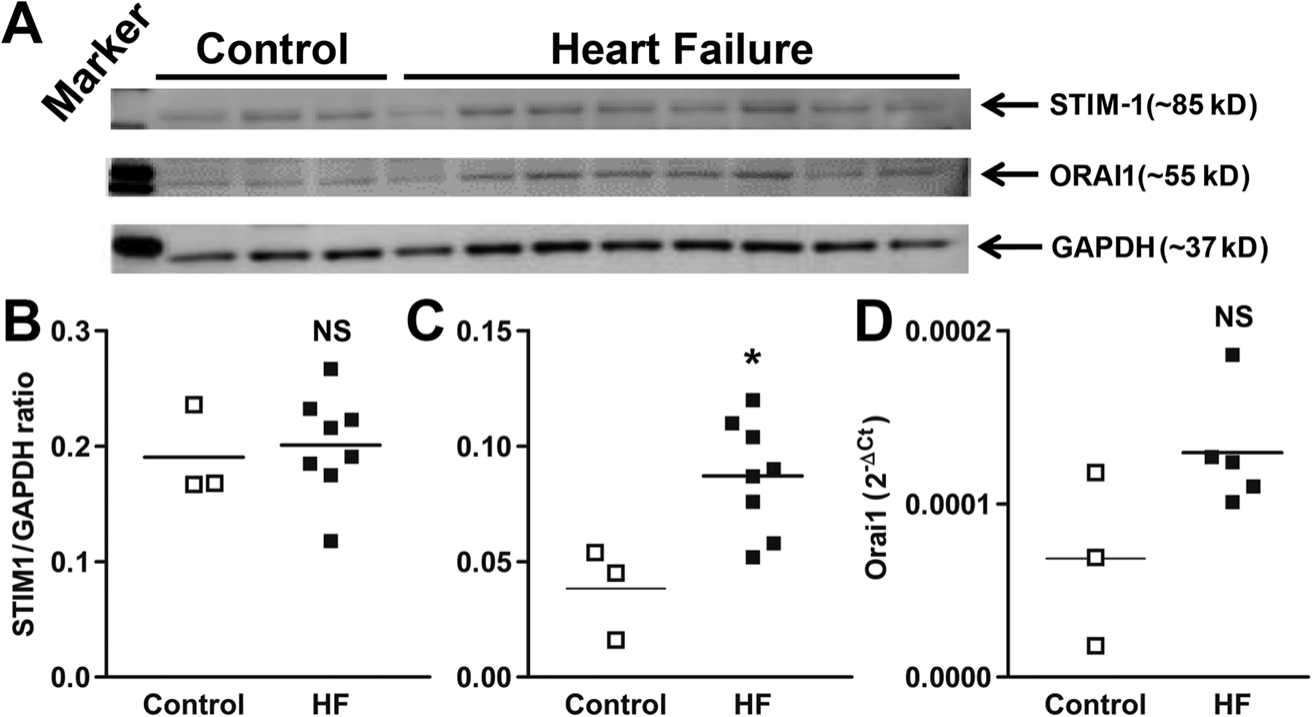
**Expression of I_CRAC_–channel components, STIM1 and ORAI1.** (A) Immunoblots of hVF lysates probed for the proteins STIM1, ORAI1 and GAPDH. Enhanced expression of the ORAI1 protein (*P*<0.05) in the hVFs isolated from failing hearts compared with the healthy hearts (middle) but no change in STIM1 (top). (B) Individual data points column graph displays the mean and each sample ratio of STIM-1 to GAPDH with no change between the groups. (C) Column graph depicts the mean and each sample ratio of ORAI1 to GAPDH with significant increase in ORAI1 expression in heart failure. *P≤0.05, unpaired *t*-test (two-tailed); control *n*=3, HF *n*=8. (D) Real time-PCR data (2^−ΔCt^) in individual data points column graph shows a tendency (*P*=0.08) toward increased mRNA expression (1.89-fold) of *ORAI1* gene in failing heart hVFs. Unpaired *t*-test (two-tailed); control *n*=3, HF *n*=4. NS, not significant.

Among different SOC entry processes, the predominant mechanism is via the pore-forming ORAI1 multimers, regulated by interactions with endoplasmic reticulum Ca^2+^ sensor, stromal interaction molecule 1 (STIM1) (Chen et al., 2010; Prakriya et al., 2006). Therefore, to elucidate the underlying mechanisms in the enhanced Ca^2+^ influx of hVF-HFs, we examined the protein expression levels of ORAI1 and STIM1 in the hVF-HF and control groups. Both ORAI1 and STIM1 protein bands were detectable at their expected sizes of 55 kDa and 85 kDa, respectively, and were compared after normalization to GAPDH (37 kDa). While there was no significant difference in STIM1 protein expression (0.19±0.02 vs 0.2±0.02, *P*=0.73; control *n*=3, hVF-HF *n*=8; Fig. 5A,B), the expression of ORAI1 was significantly greater in the hVF-HF group (0.09±0.008, *n*=8) than the control group (0.04±0.01, *P*=0.01, *n*=3; Fig. 5A,C). Because the protein expression of ORAI1 is enhanced in failing heart ventricular fibroblasts, we used RT-PCR to determine whether the enhanced expression occurred at the transcriptional level. No significant difference in the gene expression of *STIM1* existed, while a trend toward higher *ORAI1* gene expression in the hVF-HF group than the control group was present (*P*=0.08; Fig. 5D).

### Co-localization of ORAI1 and STIM1 is high in ventricular fibroblasts from failing hearts

**Fig. 6.**
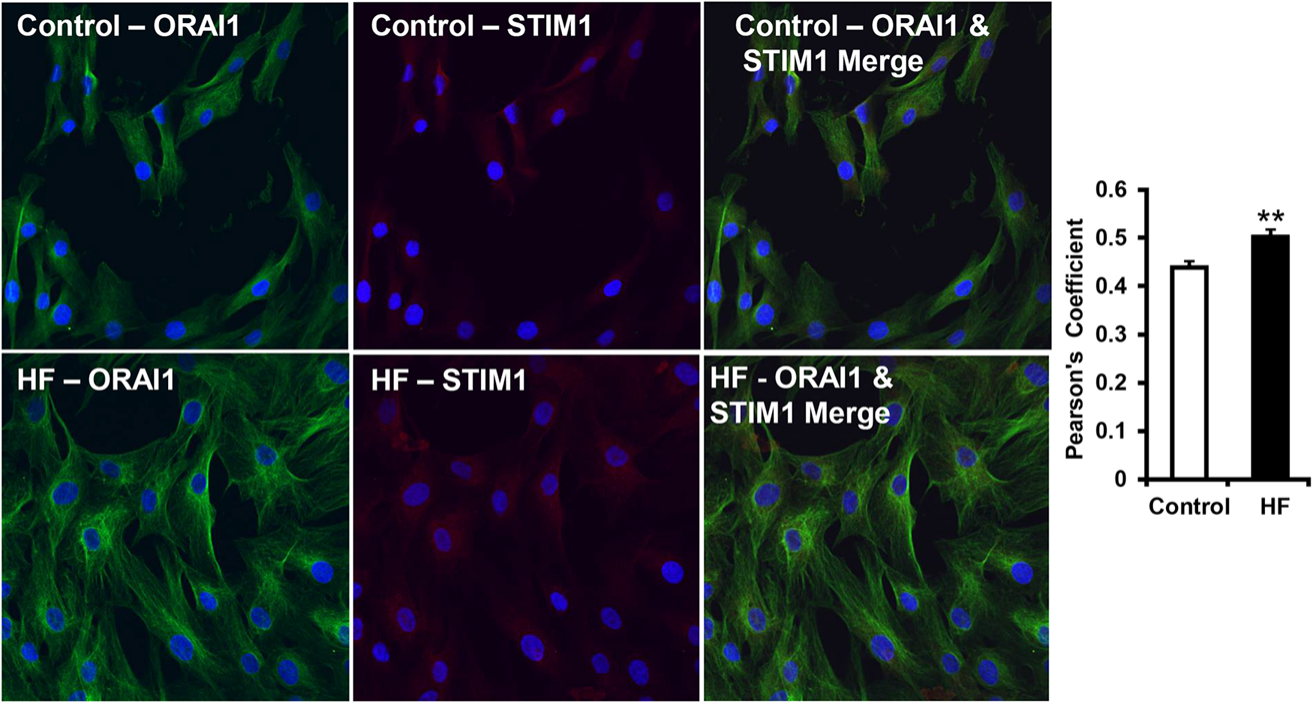
**Immunolabeling and co-localization index of I_CRAC_-channel components, STIM1 and ORAI1.** Representative confocal microscopic images and double immunolabeling of ORAI1 (green) and STIM1 (red) from control (top panel) and failing heart human ventricular fibroblasts (bottom panel) are shown. Relatively, green fluorescence intensity (ORAI1) is greater in the failing heart hVFs versus control hVFs. The co-localization of ORAI1/STIM1 (right images), calculated by Pearson's correlation co-efficient using the Fluoview software, is significantly (**P=0.001) high in failing heart ventricular fibroblasts (*n*=63 cells from three randomly selected, different heart failure patients), compared with non-failing heart control hVFs (*n*=181 cells from three randomly selected different normal individuals) (right bar graph). Data are presented as mean±s.e.m., analyzed by unpaired *t*-test (two-tailed).

Using confocal microscopy, we determined whether there was increased co-localization of the pore-forming ORAI1 multimers and the regulatory endoplasmic reticulum Ca^2+^ sensor, STIM1. Confocal microscopic images and double immunolabeling of ORAI1 (green) and STIM1 (red) from the control and hVF-HF groups are shown in Fig. 6. Using Fluoview software (version 4.2a; Olympus, Melville, NY), we calculated the Pearson's correlation coefficient for co-localization of ORAI/STIM1, which was significantly high in the hVF-HF group (0.5±0.01; *n*=63 cells from three different patients) compared to the control group (0.4±0.01; *n*=181 cells from three different individuals, *P*<0.01; Fig. 6).

### Enhanced collagen secretion from failing heart ventricular fibroblasts is dependent on SOCE

**Fig. 7.**
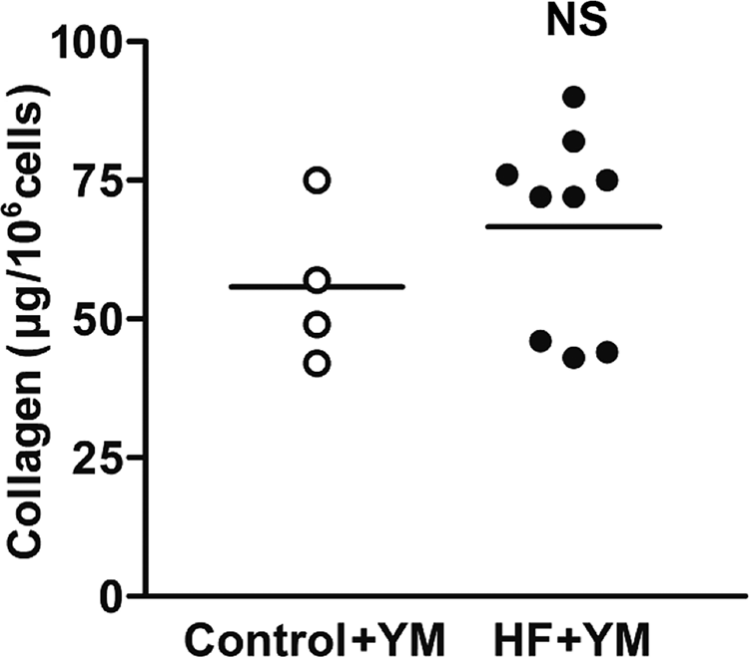
**Effect of inhibition of I_CRAC_ channels on the increased secretion of collagen by ventricular fibroblasts isolated from failing hearts.** Incubation of fibroblasts both from the failing heart and control groups with YM58483 (10 µM), a selective I_CRAC_-current inhibitor, for 48 h, eliminated the increase in collagen secretion of failing heart fibroblasts (HF+YM) compared to non-failing heart fibroblasts (control+YM) as shown in the individual data points column graph with mean and each sample value for each group. Overall, the percentage of inhibition by the presence of YM58483 of the collagen secreted/deposited from failing heart and normal heart hVFs in the absence of the drug was ∼17% and ∼5%, respectively. Data were analyzed by unpaired *t*-test (two-tailed); NS, not significant; control *n*=4, HF *n*=11. *P*≤0.05 was considered significant.

To determine if elevated ORAI1 expression and SOCE in hVF-HFs were responsible for greater collagen secretion, we assessed the impact of selective pharmacological inhibition of I_CRAC_ on collagen secretion and compared it with control hVFs. As displayed in Fig. 7, 48-h incubation of fibroblasts with YM58483 (10 µM), a selective I_CRAC_-current inhibitor (Ohga et al., 2008; Yoshino et al., 2007; Zitt et al., 2004), eliminated the increase in collagen secretion in the hVF-HF group compared with the control group (66.78±5.87 µg/10^6^ cells vs 55.81±7.09 µg/10^6^ cells, *P*=0.27; *n*=4-7). The overall percentage of inhibition by YM58483 of the amount of collagen secreted/deposited in the absence of the drug was about 5% in the control fibroblasts and 17% in failing heart fibroblasts, indicating a greater role of I_CRAC_-mediated Ca^2+^ influx in enhanced collagen secretion by failing heart hVFs.

## DISCUSSION

The overall findings of this study in human failing hearts compared with non-failing hearts are: (1) enhanced cardiac fibrosis, (2) increased collagen secretion by isolated fibroblasts, (3) elevated Ca^2+^ influx via SOCE in isolated fibroblasts, (4) augmented expression of the ORAI1 channel, the pore-forming component of the I_CRAC_ channel, (5) no change in the store Ca^2+^ sensor, STIM1 expression, (6) increased co-localization of STIM1/ORAI1, and (7) I_CRAC_ channels as a mediator of enhanced collagen secretion, which can be suppressed by pharmacological channel inhibition.

Heart failure in the elderly is one of the leading causes of morbidity, mortality, hospitalization, and increased healthcare utilization (Mozaffarian et al., 2016). Most of the focus of cardiac research in heart failure has been on cardiomyocyte function; little attention has been given to the ECM production that plays an equally important role in the pathogenesis of heart failure and its progression (Hinz, 2015). Despite this recognition, insights into the molecular mechanism underlying excessive fibrosis have been limited and no specific therapies primarily targeting cardiac fibrosis have been approved. This is mainly because a complete understanding of the fibrogenesis and regulatory mechanisms involved in excessive ECM deposition by fibroblasts – which reduces tissue compliance and contributes to progression of heart failure – is lacking. Therefore, the objective of our study was to identify molecular mechanisms underlying excessive production of collagen in fibroblasts from human failing hearts.

In histology of tissues obtained from the left ventricle of patients undergoing LV assist device implantation for advanced heart failure, excessive fibrosis was present more often than in tissues from non-diseased hearts, and this was associated with an increased ECM deposition capacity of fibroblasts from the failing heart. To elucidate the mechanism underlying increased secretion/deposition of collagen by the failing heart, the intracellular calcium dynamics, one of the main mediators of collagen secretion (Janssen et al., 2015), was compared between hVF-HF and the control. In fibroblasts, unlike excitable cells that require calcium influx through voltage-operated calcium channels, the major sources of Ca^2+^ influx are through SOCE pathways that are activated by depletion of intracellular calcium stores, such as within the endoplasmic reticulum. I_CRAC_ (Ca^2^^+^-release-activated channel) (Chen et al., 2010) is one such pathway and has two components: (1) a pore-forming channel subunit formed by ORAI1 localized in the plasmalemma and (2) a regulatory subunit within the endoplasmic reticulum, STIM1, that senses calcium depletion when it loses the Ca^2+^ from its Ca^2+^-binding EF hand, and, through its conformational changes and redistribution toward the proximity of plasmalemma, activates ORAI1 to allow calcium entry into the cells (Trebak et al., 2013). HVFs from failing and non-failing hearts demonstrated a similar response to thapsigargin, an inhibitor of the sarcoplasmic endoplasmic reticulum Ca^2+^-ATPase (SERCA) that is used to deplete endoplasmic reticulum calcium with a resultant elevation in cytoplasmic calcium. This is unlike studies in failing heart cardiomyocytes, in which altered Ca^2+^ levels caused by reduced sarcoplasmic reticulum Ca^2+^ uptake was evident because of de-phosphorylation of phospholamban (Reuter and Schwinger, 2012; Schwinger et al., 1999). In our study, the subsequent Ca^2+^ influx via SOC channels was significantly elevated in fibroblasts from failing hearts more so than in those from non-failing hearts. These results in hVFs from patients with advanced heart failure are similar to the previous observations by Nattel's Laboratory in atrial fibroblasts from dogs with ventricular tachypacing-induced heart failure (Qi et al., 2015), suggesting the SOCE pathway may play a major role in promoting fibrosis in heart failure and that this response is cardiac-chamber independent and observed in a canine model as well as human patients with advanced heart failure. The elevated calcium influx through the I_CRAC_ channel was related to functional and translational changes in the ORAI1 pore-forming subunit of the I_CRAC_ channel that was ∼2.2-fold elevated in the hVF-HF group compared with the control group without any significant change in STIM-1 protein subunit expression. Messenger RNA for both the regulatory and pore-forming subunits of the I_CRAC_ channel was not significantly different between fibroblasts from failing and non-failing hearts. These observations suggest that the increased SOC influx in hVF-HFs is mainly caused by an increase in the expression of the ORAI1 subunit through post-transcriptional changes. The mechanism underlying selective upregulation of the ORAI1 subunit is not known, but the post-transcriptional expression of the *ORAI1* gene has been shown to be regulated by miR-519 using TargetScan/complementarity studies in HELA cells (Abdelmohsen et al., 2012). Whether similar regulation by MIR-519 occurs in hVF-HF is not known, but in our studies we were unable to detect MIR-519 expression in fibroblasts from non-failing or failing hearts, which is suggestive that other possible regulatory mechanisms are involved (Sauc et al., 2015). The greater expression of ORAI1 may increase the channel availability by immobilization of mobile CRAC channels, trap the tetrameric ORAI1 channels (Hoover and Lewis, 2011), and transition ORAI1 channels to a high-open probability (Po) state, which is normally dependent on the STIM1: ORAI1 stoichiometry and relative locations and abundance of STIM1 and ORAI1 (Hoover and Lewis, 2011). Indeed, double immunolabeling confocal images show that the Pearson's correlation coefficient for co-localization of ORAI1 and STIM1 is significantly high in the hVF-HF group compared with the control group. The expression of the genes *COL1A1* or *COL1A2,* which encode for collagen proteins, was not significantly different between the hVF-HF and control groups, and thus, increased collagen was related to enhanced secretion from fibroblasts that were sensitive to inhibition by a specific pharmacological inhibitor of the I_CRAC_ channel.

The I_CRAC_ channel is associated with cellular calcium signaling that has been well characterized in cells involved in inflammation and immunity, pulmonary artery smooth muscle cells from patients and animal models with pulmonary arterial hypertension (Fernandez et al., 2015), and in cardiomyocytes (Voelkers et al., 2010). An inhibitory effect of ORAI1 knockdown on cardiomyocyte hypertrophy and pro-hypertrophic signaling was demonstrated in neonatal ventricular cardiomyocytes (Voelkers et al., 2010). Although ORAI1 and STIM1 have been described in fibroblasts and human cardiac fibroblasts (Almirza et al., 2012; Chen et al., 2010), their role in fibrosis in patients with advanced heart failure has not been described. Therefore, the current study in hVFs demonstrating a role of these channels in modulating cardiac fibrosis is not only innovative, but also of great clinical significance. In studies in transgenic mice, an association was found between overexpression of STIM1, which enhanced calcium entry following intracellular store depletion, and increased diastolic calcium levels and enhanced susceptibility toward sudden death and heart failure with hypertrophy (Correll et al., 2015). Heterozygous deletion of ORAI1 resulting in 50-75% reduction in ORAI1 protein expression in mice with pressure overload cardiac hypertrophy mediated by transverse aortic constriction was associated with a detrimental effect on survival with progressive heart failure (Horton et al., 2014). These observations strongly support the role of ORAI1 in cardiomyocytes of generating a compensatory response to pressure overload and at the same time regulating cardiac fibrosis (Ichiki et al., 2014). The increased collagen secretion seen in the hVF-HFs was sensitive to selective inhibition of I_CRAC_ channels by YM58483, a selective inhibitor of I_CRAC_ channels (Ohga et al., 2008; Yoshino et al., 2007), providing additional support for its role in mediating calcium signaling underlying the increased secretion of collagen and ECM by fibroblasts in the failing heart. This is of great clinical significance as augmented SOC-Ca^2+^ influx in fibroblasts could be targeted to reduce ECM deposition, an important factor that contributes to progressive fibrosis, reduced myocardial compliance, and increased stiffness accelerating progression of heart failure and the development of the substrate for arrhythmogenesis (Spadaccio et al., 2015).

In conclusion, fibroblasts from the failing ventricles had an increased capacity for collagen secretion compared with fibroblasts from non-failing hearts, and this is related to an upregulation of SOCE associated with enhanced expression of ORAI1, the pore-forming subunit of the I_CRAC_ channel. This mechanism of augmented SOC Ca^2+^ influx in failing heart fibroblasts could be therapeutically exploited to limit cardiac fibrosis by selective inhibition of I_CRAC_. Additional studies preventing upregulation of I_CRAC_ activity in heart failure animal models by direct inhibition of the channel or through allosteric modulation or expression of channel proteins by modulating its expression through microRNA are warranted to further define the role of this pathway in limiting fibrosis and heart failure progression.

## MATERIAL AND METHODS

### Study population

The portion of the LV that is removed during LV assist device (LVAD) implantation was used to extract hVFs that were grown as primary culture. Patients with New York Heart Association class III to IV heart failure who underwent either cardiac transplantation or LVAD implantation were studied. Vendor-authenticated HVFs isolated from trauma victims free of any structural heart disease were used as a control (Lonza, Allendale, NJ, #CC-2904; ScienCell, Carlsbad, CA, #6310; Cell Applications, San Diego, CA, #306v-05a). The study was approved by the Institutional Review Board and adhered to the Health Insurance Portability and Accountability Act (HIPAA) and the institution's patient privacy and security guidelines. Informed consent was obtained from all subjects. The study conformed to the principles of the Declaration of Helsinki.

### Isolation of ventricular fibroblasts

Cardiac tissues were transferred from the operating theater in Dulbecco's phosphate-buffered saline (DPBS) on ice. The ventricular tissue block was carefully cleaned to remove all non-myocardial portions in a 60* *mm culture dish in the tissue culture hood and cut into 1* *mm blocks, transferred to a 25 cm^2^ TPP tissue culture flask (MidSci, St Louis, MO), washed thrice with DPBS, twice with FM-b (ScienCell) containing penicillin/streptomycin, spread evenly into 20-30 blocks per flask and cultured in 5 ml FM-2 media (ScienCell) containing 5% fetal bovine serum (FBS) and penicillin/streptomycin. The flask was inverted, with the bottom (surface with tissue) up, in the incubator (37°C; 21% O_2_; 5% CO_2_) and blocks were allowed to adhere for about 4 h. After 2-3 h, the flasks were turned to the normal orientation. The media was changed every 2 days. In 2 weeks, cardiac fibroblasts migrated from the ventricular tissue explants and reached 70% confluency. The cells were trypsinized and transferred to 150 cm^2^ TPP tissue culture flasks (MidSci) with FM-2 media (ScienCell) containing 5% FBS and penicillin/streptomycin and grown to 70% confluency. These initial cultures were split, and passages 2 and 3 were stored in liquid nitrogen until experiments were conducted.

### Histology of the left ventricle

Pieces of LV tissue were washed in phosphate-buffered saline (PBS) fixed overnight in 4% paraformaldehyde, and embedded in paraffin by standard protocols (Wolf et al., 2005). Following serial sectioning, 5 μm sections were stained with Masson's trichrome. The fibrosis area within sections was measured by observers blinded to the groups by visualizing blue-stained areas. Using ImageJ software (http://rsbweb.nih.gov/ij/), blue-stained areas and non-stained myocardial areas from each section were determined using color-based thresholding. The percentage of total fibrosis area was calculated as the summed blue-stained areas divided by total myocardial area.

### Collagen assay

Ventricular fibroblasts (passage 3 or 4) from both failing and non-failing hearts were plated separately at 10^4^ cells/cm^2^ in T-25 flasks in FM-2 media (ScienCell) containing 5% FBS. After 24 h, the media was replaced with fresh media with or without YM-58483 (10 µM), a specific blocker of I_CRAC_ channel (Ohga et al., 2008; Yoshino et al., 2007; Zitt et al., 2004). After 48 h, the soluble collagen deposited in the ECM was quantified colorimetrically using Sircol™ soluble collagen assay kit (Biocolor) and normalized to number of cells for analysis. The assay was performed in all the samples at the same time and repeated at least twice.

### Ca^2+^ imaging

Ventricular fibroblasts from both failing and non-failing hearts were plated at 10^4^ cells/cm^2^ in collagen-coated MatTek dishes (MatTek Corp., Ashland, MA) in FM-2 media containing 5% FBS. After 24 h, the media was replaced with serum-free fresh media. After 18-24 h of serum-starvation, the cells were incubated in FM2 media containing fluo-3 dye (2 µM) for 30 min. Cells were washed with Ca^2+^-free DPBS twice and kept in Ca^2+^-free Krebs-Ringers Henseleit (KRH) solution that contained (in mM): NaCl, 116; KCl, 4; MgCl_2_, 1; glucose, 25 and HEPES, 10 (pH 7.4). Under confocal microscopy (Olympus IX83 microscope, Fluoview Ver 4.2a software), in Ca^2+^-free KRH, thapsigargin (2.5 µM) was added to release the Ca^2+^ from intracellular stores and deplete the store. CaCl_2_ (2 mM) was then added extracellularly to facilitate Ca^2+^ influx via the store-operated channels that opened because of Ca^2+^ store depletion. Ionomycin (2 µM), a Ca^2+^ ionophore, was added to determine the maximum possible Ca^2+^ fluorescence in the selected region of interest, which was used for normalization of the test Ca^2+^-induced fluorescence. Peak fluorescent intensities following thapsigargin, CaCl_2_, or ionomycin were captured and average values from at least two sets of cells per patient or control were obtained for analysis. Data were analyzed as the percentage of ionomycin response for each cell.

### Immunoblotting of STIM1 and ORAI1 proteins

Standard western blotting protocols were followed per the manufacturer's protocol. Equal amounts of proteins were loaded and electrophoresis was performed at 110 V for 2 h. The separated proteins were transferred to PVDF membrane (7 min iBlot) and probed for STIM1 and ORAI1 proteins using STIM1 (D88E10) monoclonal antibody (#5668, Cell Signaling) and monoclonal Anti-ORAI1 antibody (#SAB3500126, Sigma), respectively. All samples were subjected to immunoblotting simultaneously; this was repeated at least twice.

### RT-PCR of *COL1A1*, *COL1A2*, *STIM1* and *ORAI1* genes

Total RNA was isolated from fibroblasts using Trizol Reagent (Thermo Fisher Scientific) according to the manufacturer's instructions. RNA concentrations were evaluated spectrophotometrically using the NanoQuant on the Tecan Infinite M200 plate reader (Tecan Group, Mannedorf, Switzerland). Total RNA (300 ng) was used for reverse transcription using the miScript RT II kit with the supplied HiFlex buffer (Qiagen). qPCR was performed on the ABI 7300 Real-time PCR System (Applied Biosystems, Thermo Fisher Scientific), using the Power SYBR Green PCR Master Mix (Thermo Fisher Scientific) and 10 ng diluted cDNA per well. The following primers were used: *COL1A1* (NM_000088.3) R: 5′-AGCCTCTCCATCTTTGCCAGCA-3′, F: 5′-GATTCCCTGGACCTAAAGGTGC-3′; *COL1A2* (NM_000089.3) R: 5′-ATCACCACGACTTCCAGCAGGA-3′, F: 5′-CCTGGTGCTAAAGGAGAAAGAGG-3′; *STIM1* (NM_003156) R: 5′-CTGTCACCTCGCTCAGTGCTTG-3′, F: 5′-CACTCTTTGGCACCTTCCACGT-3′; *ORAI1* (NM_032790) R: 5′-GACCGAGTTGAGATTGTGC-3′, F: 5′-GAGTTACTCCGAGGTGATGA-3′; *B2M* (NM_004048) R: 5′-CCAATCCAAATGCGGCATCTTCA-3′, F: 5′-CCACTGAAAAAGATGAGTATGCCT-3′. The cycling conditions were 50°C for 2 min, 95°C for 10 min, followed by 40 cycles at 95°C for 15 s, 1 min at 60°C, and 72°C for 40 s. Melt curve analysis was performed by an additional dissociation step of 1 cycle at 95°C for 15 s and ramping data collection at 60°C for 1 min and 95°C for 15 s. Ct values were calculated using Applied Biosystems 7300 system software v1.4.0. Data were normalized against the β-2-microglobulin (*B2M*) reference gene. Relative expression values were obtained by normalizing Ct values of the tested genes with Ct values of the reference gene, using the ΔCt method.

### Immunolabeling of STIM1 and ORAI1

HVFs were plated in collagen-coated MatTek dishes in FM-2 media containing 5% FBS. After 18-24 h, media was replaced and incubated for 2 h. To initiate store-Ca^2+^ depletion, hVFs were treated with thapsigargin (2.5 µM) for 15 min. HVFs were then fixed in 4% paraformaldehyde at 4°C for 20 min and permeabilized with 0.2% (v/v) Triton X-100 in PBS for 45 min and blocked with 10% (v/v) donkey serum (Sigma) containing 0.2% (v/v) Triton X-100 for 45 min. After rinsing in HBSS, the samples were incubated with primary antibodies – rabbit monoclonal anti-STIM1 (D88E10) antibody (#5668, Cell Signaling), and mouse monoclonal anti-ORAI1 antibody (#SAB3500126, Sigma) – and diluted (1:200) in 1% donkey serum with 0.2% Triton X-100/PBS overnight at 4°C. Samples were washed with DPBS (no Ca^2+^/Mg^+^) three times and incubated with secondary antibodies conjugated with Alexa Fluor 594 (anti-rabbit IgG) or Alexa Fluor 488 (anti-mouse IgG) in 1% (w/v) donkey serum with 0.2% (v/v) Triton X-100 in PBS. After washing three times with DPBS, two drops of NucBlue Fixed cell stain (Molecular Probes, # R37606) were added to stain the nucleus for 5 min at room temperature. After washing again with DPBS twice and HBSS (with Ca^2+^/Mg^+^), confocal images were acquired with the Olympus confocal microscopy system.

### Data analysis

Comparisons of mean values between control and hVF-HF groups were performed by unpaired *t*-test. *P*≤0.05 was considered statistically significant. Sample sizes for Ca^2+^-imaging and collagen-secretion assays were calculated using University of California – San Francisco Clinical & Translational Science Institute calculators (http://www.sample-size.net/sample-size-means) with 80% statistical power.
